# Type 2 diabetes mellitus in children and adolescents: a relatively new clinical problem within pediatric practice

**Published:** 2016

**Authors:** OR Temneanu, LM Trandafir, MR Purcarea

**Affiliations:** * Mother and Child Department, “Grigore T. Popa” University of Medicine and Pharmacy, Iasi, Romania; ** Faculty of Medicine, “Carol Davila” University of Medicine and Pharmacy, Bucharest, Romania

**Keywords:** : type 2 diabetes mellitus, obesity, insulin resistance, children, adolescent

## Abstract

Type 2 diabetes mellitus is a complex, chronic metabolic disease, presents a heterogeneous etiology, with risk factors at the social level and behavioral, environmental, and genetic susceptibility. It is associated with serious complications, but the early diagnosis and initiation of therapy may prevent or delay the onset of long-term complications. In children and adolescents, it was observed in particular increasing the prevalence of T2DM along with obesity, which is associated with insulin resistance. Patient and family education for a young person with T2DM is very important and will focus on behavioral changes (diet and activity).

## Introduction

The alarming increase in the incidence of obesity in children and adolescents worldwide determined the increased risk for the occurrence of comorbidities, its injury being cardiovascular disease, type 2 diabetes mellitus (T2DM), metabolic syndrome, non-alcoholic fatty liver disease (NAFLD), osteoarthritis, obstructive sleep-apnea (OSA), some forms of cancer [**1**]. 

DM is a complex, chronic metabolic disease, with a heterogeneous etiology and risk factors at the social level and behavioral, environmental, and genetic susceptibility. It is associated with serious complications, but the early diagnosis and initiation of therapy may prevent or delay the onset of long-term complications. Chronic complications of diabetes include the development of cardiovascular disease, end-stage kidney disease, retinopathy leading to blindness and limb amputations. All these complications contribute to excess morbidity and mortality in patients with diabetes mellitus. The new management strategy helps children with T2DM live a long and healthy life.

## Short history

Diabetes has been affecting human life for thousands of years. In manuscripts dating from 1550 BC, the Egyptians described a disease suspected to be diabetes. According to The National Medical Journal of India, the ancients were familiar with the disease, as the testing method was using ants if a person was suspected of suffering from the so-called disease “sweet urine”. In Greek, “diabese” means “pass through”. Historical documents showed that Greeks, Persians, Chinese, Japanese, and Indians were aware of this pathology, but no one could determine the cause. Thus, a diagnosis of diabetes was most likely associated with a death sentence. The American Diabetes Association (ADA) reported that in 1910, health professionals have taken the first steps towards finding the causes and treatment for diabetes. Edward Albert Sharpey-Schafer announced that the pancreas of a patient diagnosed with diabetes was unable to produce what he called “insulin”. On June 21, 1921, the Romanian scientist, Nicolae Paulescu (1869-1931), discovered a hormone in the pancreas of animals, which he called “Pancreine”. In 1921, two Canadian researchers, Frederick Grant Banting and Charles Herbert Best, have successfully extracted insulin from healthy dogs, which they injected later in dogs with diabetes. The medical condition has improved considerably. According to his writings, which were published by his son Richard in Diabetic Medicine, Harold Himsworth finally distinguished between the two types of diabetes in 1936. He defined them as “insulin-sensitive” and “insulin-insensitive”. Today, these classifications are commonly referred to as “Type 1” and “Type 2” Diabetes. For many years, T2DM has not been crowned by therapeutic successes. Antidiabetics were developed only in the 1950s [**2**].

According to the latest statistics from the International Diabetes Federation (IDF), Type 1 DM affects approximately 500,000 children aged below 15 worldwide. Only in 2013, there were 79,000 diagnosed cases of type 1 diabetes in children, which means an annual increasing incidence of 3%. The big challenge in taking care of the child with diabetes is represented by the increased incidence of type 2 diabetes among children and adolescents.

30 years ago, T2DM was considered rare in children and adolescents. However, in the mid 1990s, researchers began to notice a growing incidence of T2DM worldwide [**3**]. This was particularly observed in the United States, but has also been reported in other countries, such as Canada, Japan, Austria, Britain and Germany. In some regions of the United States, T2DM is as common in adolescents as type 1 DM. This observation followed the one that concentrated upon the increasing prevalence and incidence in obesity and overweight in children and adolescents [**4**]. 

Although T2DM is widely diagnosed in adults, the frequency has increased significantly in the pediatric age group at the end of the twentieth century. Depending on the study population, type 2 diabetes now accounts for 8-45% of all new cases of diabetes reported among children and adolescents [**5**]. The SEARCH for Diabetes in Youth Study (observational multicenter study conducted in the United States) found that the incidence of type 2 diabetes was the highest among American Indians aged 15-19 years (49.4 cases per 100,000); the second and third highest incidence belonged to the black race Asians and islanders of the Pacific, aged 15-19 years, with 22.7 cases per 100,000 people respectively 19.4 cases per 100,000 people [**6**]. An increased prevalence of T2DM has also been recognized in countries other than the United States, including Japan, where the incidence of T2DM in children of school after 1981 proved to be closely related to a growing prevalence of obesity. Studies among various countries (China, Taiwan, Bangladesh, Australia) have also shown an increased incidence of T2DM [**7**]. 

According to the statistics provided by the Romanian Society of Diabetes, Nutrition and Metabolic Diseases regarding Romania, in 2014, 376 new cases of diabetes in children were registered nationally, 16 of which were cases of T2DM. Overall, there are 2,670 Romanian children with diabetes among the doctors’, pediatricians’ and diabetologists’ records [**8**].

The prevalence of T2DM in children and adolescents is higher among girls than boys, as the prevalence is higher among adult women than adult men [**9**]. 

## Etiopathogenesis

The causes of T2DM are multiple and consist of a combination of genetic predisposition with current lifestyles: nutrition, physical inactivity, marketing, and media influences, which are characteristic for the contemporary obesogenic environment. The importance of the hereditary component etiopathogeny type 2 diabetes is supported by an increased prevalence in the first-degree relatives of patients with T2DM, high concordance in monozygotic, increasing prevalence in certain ethnic groups.

Also, obesity, the most important risk factor in developing T2DM in young people, is closely correlated with an increasing number of cases of T2DM [**10**].

The analysis of pancreatic beta cells from diabetic and healthy individuals revealed epigenetic changes in approximately 850 genes with a fold change between 5-59%. Though many genetic variants are shown to contribute to the development of T2DM, to date only PPARG, KCNJ11 and TCF7L2 are established genes associated with common forms of T2DM [**11**].

There are a number of rare cases of diabetes that arise due to an abnormality in a single gene (known as monogenic forms of diabetes). These include among others, maturity onset diabetes of the young (MODY), Donohue syndrome and Rabson-Mendenhall syndrome [**12**].

An increase in the prevalence of T2DM along with obesity, which is associated with insulin resistance, was observed particularly in children and adolescents. Insulin resistance is a common manifestation of obesity and is associated with T2DM hyperlipidemia, hypertension, acanthosis nigricans, ovarian hyperandrogenism, and NAFLD. Initially, pancreatic beta cells are able to compensate the IR by increasing the secretion of insulin in the pathogenesis of glucose intolerance. The compensatory hyperinsulinemia induces an increased appetite and weight gain. After the pancreatic beta cells function declines and an insufficient secretion of insulin appears, a transition will be caused from the stage of insulin resistance to impaired glucose tolerance, followed by T2DM. 

Most often, the disease is diagnosed around the age of 13-14 years, with an earlier onset in girls, suggesting that the physiological insulin resistance during puberty may play an important role.

The main risk factors for T2DM in children and adolescents are obesity combined with genetic predisposition and/ or family history in addition with children born small for a gestational age (< 2500 g), newborn macrosomia of diabetic mother (> 4000 g), premature adrenarche in girls (pubic hair appearing before the age of 8 years). An increase in the metabolic disease and hypertension was reported in males with a lower birth weight, which supports the theory of programming conditions from the intrauterine life [**13**,**14**].

On the other hand, intestinal microbiota influences the development of conditions characterized by chronic low-level inflammation, such as obesity and T2DM, through systemic exposure to bacterial lipopolysaccharide derived from the intestinal microbiota [**15**].

## Diagnostic criteria for T2DM in childhood and adolescence

Diagnostic criteria for diabetes are based on blood glucose measurements and the presence or absence of symptoms (American Diabetes Associations, 2015) [**16**]:

• a fasting plasma glucose (FPG) > 126 mg/ dL (7.7 mmol/ l)

- on several occasions if without symptoms (polyuria, polydipsia, weight loss)

• a random plasma glucose sample ≥ 200 mg/ dL (11,1 mmol/ l)

- on several occasions if without symptoms (polyuria, polydipsia, weight loss)

• 2 hrs post glucose challenge ≥ 200 mg/ dL (11,1 mmol/ l) 

performed with 1,75 g glucose/ kg, max 75 g glucose dissolved in water

• Hb A1c ≥ 6,5% (48 mmol/ l) – if tested in a certified lab.

However, HbA1C as a sole marker to diagnose DM is still controversial at present.

Children with T2DM can also present classical diabetes symptoms such as polyuria, polydipsia, blurred vision, and weight loss, in association with glycosuria and, in some cases, ketonuria. T2DM occurs occasionally with diabetic ketoacidosis or hyperosmolar nonketonic crisis at presentation, which can be fatal [**17**]. 

T2DM is often asymptomatic in children and adolescents. In the absence of symptoms or presence of mild symptoms of diabetes, hyperglycemia detected incidentally or under conditions of acute infection, traumatic, circulatory, or other stress may be transitory and should not be regarded as a diagnosis of diabetes itself. In the absence of symptoms, the diagnosis of diabetes should not be based on a single plasma glucose concentration.

In 2015, the American Diabetes Association recommended screening for T2DM in children with Overweight (BMI > 85th percentile for age and sex, weight for height > 85th percentile, or weight > 120% of ideal for height) with 2 additional risk factors for T2DM:

• family history of type 2 diabetes in first - or second-degree relative 

• race/ ethnicity (Native American, African American, Latino, Asian American, Pacific Islander)

• signs of insulin resistance or conditions associated with insulin resistance: acanthosis nigricans, hypertension, dyslipidemia, polycystic ovary syndrome, or small for-gestational-age birth weight

• maternal history of diabetes or gestational diabetes mellitus during the child’s gestation [**16**].

The age of screening initiation for T2DM in children is 10 years or at the onset of puberty, if puberty occurs at a younger age. Periodic retesting should be undertaken at every 3 years, until the diagnosis is established or refuted.

It is necessary to distinguish between T1DM and T2DM, in the case of a patient who was newly diagnosed with diabetes at the beginning of the assessment. Clinical signs helpful in distinguishing T2DM from T1DM are obesity and signs of insulin resistance. The differences between adult and pediatric T2DM are represented in **Table 1**. Patients with T2DM frequently have elevated C-peptide levels. The absence of insulin autoantibodies, the islet cell, and/ or glutamic acid decarboxylase is also typical.

**Table 1:** The differences between adult and pediatric T2DM

Parameters	Adults	Children and adolescents
Age	> 40 years	> 10 years
Onset	insidious	insidious/ signs of hyperglycemia
Sex	both	predominantly female
Pancreatic beta cells function declines	insidious	faster (under 4 years)
Treatment	lifestyle modification	lifestyle modification
	various types of oral antidiabetic agents	Metformin+/ - Insulin
Complications/ comorbidities	late	early (after approx 2-2.5 years after onset)

The earlier onset of T2DM leads to an earlier onset of complications (progressive neuropathy, retinopathy leading to blindness, nephropathy leading to chronic renal failure, atherosclerotic cardiovascular disease). Therefore, the early diagnosis and intensive treatment is very important.

The recommendations for the specific testing of comorbidities and complications of T2DM in young people are the following:

• testing for albuminuria should be performed at the time of diagnosis and annually thereafter;

• blood pressure should be monitored at every visit;

• testing for dyslipidemia should be performed soon after the diagnosis when blood glucose control has been achieved and annually thereafter;

• evaluation for NAFLD should be done at diagnosis and annually thereafter

• inquiries about puberty, menstrual irregularities, and OSA should be made at diagnosis and regularly thereafter;

• examination for retinopathy should be performed at diagnosis and annually thereafter [**16**].

**The management of T2DM in children and adolescents**

The treatment of T2DM in children and adolescents needs to focus on the reduction of complications. There are a few studies regarding children with T2DM, but the data provided suggests that the tight glycemic control reduces the risk of microvascular complications. 

The therapeutic goals in T2DM (American Diabetes Associations) are the following:

• weight loss;

• increase in exercise capacity;

• normalization of glycemia;

• control of comorbidities, including hypertension, dyslipidemia, nephropathy, and hepatic steatosis [**16**].

Patient and family education for young persons with T2DM is very important and will focus on behavioral changes (diet and activity). Most clinical evidence regarding T2DM in children, involves lifestyle changes (increased physical activity and changed dietary intake) and the use of only Metformin, or with insulin as well. The education and treatment team for T2DM should ideally include a nutritionist, psychologist, and/ or social worker. Therapy of adolescents with T2DM should aim to achieve fasting glucose levels under 126 mg/ dL and an HbA1c level under 6,5% within 3-4 months.

The pharmacological therapy will be used while combined with some lifestyle changes and its aim is to decrease insulin resistance, increase insulin secretion, slow down the absorption of glucose fed state. Most of the therapeutical lines recommended for treatment in children with T2DM are extrapolated from the current experience in adults.

Biguanide metformin is the most widely used worldwide. The Food and Drug Administration (FDA) approved Metformin for use in adults with type 2 diabetes in 1994. It became one of the most widely prescribed agents for this disease. In December 2000, the FDA approved the use of metformin for children aged 10 years or older, diagnosed with T2DM [**19**,**20**]. 

Metformin acts primarily by inhibiting hepatic gluconeogenesis and lowering the basal glucose. Monotherapy with Metformin can induce a fall in HbA1c averaged 1.5 percent. Tolerance is generally good, the most common side effects being the gastrointestinal ones (e.g. diarrhea, nausea, abdominal pain) [**21**]. Monotherapy with Metformin is not accompanied by hypoglycemia or weight gain.

American Academy of Pediatrics recently published the guide of the treatment’s management for T2DM in pediatric age [**22**]. The objective is the normalization of HbA1c and also the control of comorbidities (hypertension, dyslipidemia), which is very important. The ultimate goal of the treatment is to reduce the risk of acute and chronic complications associated with diabetes [**17**,**18**].

Metformin is currently the only approved oral antidiabetic in pediatric use in Europe and is recommended for children over the age of 10. Therapy starts when the children are aged 10-16 years with 500 mg/ day (one tablet = 500 mg), which can be increased to 500 mg at every 1-2 weeks, depending on the glycemic profile, until a maximum dose of 2000 mg is reached [**23**].

The clinical criteria that suggest the need for the initiation of insulin therapy in diabetes are dehydration, ketosis, and acidosis. The advantages of oral antidiabetics include:

- higher compliance of patients with the treatment

- the initial anorexic effect leading to weight loss

- reduced risk of hypoglycemia

- reduction of HbA1c by 1-2% if used for a long period of time 

- low risk of lactic acidosis 

- the amelioration of lipid metabolism with the lowering of triglycerides and LDL cholesterol.

Metformin should not be used in patients with known hypoxic disease, severe infections, liver disease, or alcohol abuse. It is also contraindicated in patients with renal failure and should be discontinued if administered in parallel with of radiocontrast substances at least 48 hours before the procedure and reinstituted only after the renal function has been proven normal. Patients receiving Metformin concomitantly with some drugs (amiloride, digoxin, morphine, procainamide, quinidine, quinine, ranitidine, triamterene, trimethoprim, and vancomycin) should be monitored for potential toxicity [**24**].

If Metformin monotherapy is not successful during the time of 3-6 months, several alternatives may be considered. Other drugs that are not approved for children and adolescents were used less frequently in children. 

Thiazolidinediones have hypoglycemic effects that increase the insulin sensitivity in liver, muscle, and adipose tissue and reduce the synthesis of hepatic glucose production. This class of drugs is not approved for pediatric use [**25**]. However, Rosiglitazone in doses of 4 to 8 mg daily was studied in a randomized trial in 2012 (TODAY study) in adolescents compared to the lifestyle changes and Metformin co-administration. Monotherapy with Metformin was associated with a durable glycemic control in about half of the patients. The association of Rosiglitazone without a major change of lifestyle was superior to the Metformin monotherapy. However, Rosiglitazone was withdrawn from the market due to its side effects and is no longer available [**26**].

As far as adults are concerned, many new drugs have been developed in the incretin domains (GLP1 analogs, DDP4 inhibitors), glitazones, glitazars, SGLT2 (and 1) inhibitors, Bromocriptine, GPR40 agonists [**14**].

## Conclusions

The primary prevention of T2DM is directed toward the obesity pandemic and involves reversing eating and entertainment trends in homes, schools, and communities that have resulted in excess caloric intake and marked decrease in energy expenditure by children and adults. Is important to comply with the recommendations of the World Health Organization about exclusively human milk until 6 months and to continue breastfeeding up to 2 years of life in the same time with complementary feeding. Regarding therapy, because Metformin is the only oral antidiabetic agent approved for pediatric use, further studies are necessary to include the new therapies used at present in adult, child and adolescent treatment. 
